# The robustness of ecosystems to the species loss of community

**DOI:** 10.1038/srep35904

**Published:** 2016-10-27

**Authors:** Qing Cai, Jiming Liu

**Affiliations:** 1 Department of Computer Science, Hong Kong Baptist University, Kowloon Tong KLN, Hong Kong

## Abstract

To study the robustness of ecosystems is crucial to promote the sustainable development of human society. This paper aims to analyze the robustness of ecosystems from an interesting viewpoint of the species loss of community. Unlike the existing definitions, we first introduce the notion of a community as a population of species belonging to the same trophic level. We then put forward a novel multiobjective optimization model which can be utilized to discover community structures from arbitrary unipartite networks. Because an ecosystem is commonly represented as a multipartite network, we further introduce a mechanism of competition among species whereby a multipartite network is transformed into a unipartite signed network without loss of species interaction information. Finally, we examine three strategies to test the robustness of an ecosystem. Our experiments indicate that ecosystems are robust to random species loss of community but fragile to target ones. We also investigate the relationships between the robustness of an ecosystem and that of its community composed network both to species loss. Our experiments indicate that the robustness analysis of a large-scale ecosystem to species loss may be akin to that of its community composed network which is usually small in size.

Ecosystems are closely related to human society. Ecosystems can be modeled as ecological networks (ENs) in which a vertex represents a species and an edge denotes the interaction between its two connecting species. To analyze ecosystems from the perspective of network science may provide new insights into the study of complex ecosystems^1^,^2^.

A long-standing quest in ecology is about the stabilities of ENs^3^,^4^. It is widely recognized that real-world ENs are nonrandom and have certain structural patterns that are essential for their stabilities^5^,^6^. Tremendous efforts have been made to investigate the underlying mechanisms or patterns that affect the stabilities of ENs^7^,^8^,^9^ (See SI text for further reading). Apart from the structural patterns of ENs, scientists also want to know to what extent ENs still can be stable if perturbations occur. As a consequence, the research direction, i.e., network robustness, has emerged and is gaining momenta.

Network robustness has been well studied^10^,^11^ in the field of conventional network science. Related work can be roughly categorized into two classes, i.e., the vertex-level robustness^12^ and the edge-level robustness^13^. The vertex (edge) -level robustness studies aim to analyze the tolerance of a network to vertices (edges) perturbations. Many researchers have shown that the robustness of a network can be enhanced by appropriately changing the network topology^12^,^14^. Studies on the robustness of ENs mainly focus on the vertex-level, such as those in refs 15 and 16. Because the topological plasticity of ENs is quite different from that of networks in common sense, studies on the robustness of ENs at the edge level have not received much attention until the venerable work in refs 17 and 18 which indicate that new interactions formed between species may enhance the robustness of ENs. A similar solid backing can be found in ref. 19 in which the authors thoroughly investigated topological plasticity and its impact on the robustness of ENs. Other studies such as those in refs 20 and 21, which explore the robustness of ENs to habitat loss and phenological change, can be regarded as mixed situations of edge-level and vertex-level network robustness, because if a habitat is lost or the phenology is changed due to the climate, species and species interactions may both go extinct. However, to the best of our knowledge, the robustness of ENs in the face of the species loss of community is little studied.

To investigate the robustness of ENs to the species loss of community, in the first place we need to better understand the notion of a community in the present context, or putting it another way, we need to find a way to identify communities from an EN. One may argue that network community detection is popular and numerous methods have been developed^22^,^23^,^24^. It should be pointed out that the existing avenues listed in refs 22, 23, 24 mainly deal with unipartite networks, whereas an EN is generally represented as a *k*-partite network. Generally speaking, there exist two possible avenues, which we refer to as the *projection* method and the *divide-and-conquer* method, to analyze the community structure of a *k*-partite network. The idea of the *projection* method is to project a *k*-partite network into a unipartite network^25^, and then apply community detection methods that are well studied and designed for unipartite networks to the projected network. This method is direct and widely used. However, in so doing, the interaction information will be lost and the discovered results may not be reliable. The *divide-and-conquer* method is to decompose a *k*-partite network into *k* − 1 bipartite networks and then analyze each bipartite network separately. The drawback of this idea is that it cannot provide a holistic view of the original network. What is more is that, most community discovery methods for bipartite networks, such as those very recent work in refs 26, 27, 28, are based on the maximization of Newman-Girvan modularity^29^,^30^ which has been proved to suffer from the curse of resolution limit^31^. It should be noted that, in ecology, a community in a trophic network, like plant-herbivore network, is also called a compartment which is a population of species containing both plants and herbivores. Compartments are appealing in ecology, because they are related to species habitats and foraging behaviors. For a *k*-partite network (*k* > 2), both the *projection* and the *divide-and-conquer* methods will classify the network into many modules each of which contains species from multiple trophic levels, for an instance, for a bee-plant-seeds feeding bird-parasitoid network, a community will contain bees, plants, seeds feeding birds, and parasitoids, which is inexplainable.

Under true scenarios, when perturbations happened due to invasive species or anthropogenic behavior, such as poisoning birds to increase crop production, species extinctions, in the beginning, may only occur among a population of species at a certain trophic level. The story of Australia’ s battle with the bunny which lasted for over a century is an example. When exotic bunnies are introduced, they will only affect the plants. Other species loss are mainly caused by secondary extinctions. With regard to this, in this paper we first put forward a new definition of community which is a group of species from the same trophic level. As a result, neither the *projection* nor the *divide-and-conquer* method works under this context, because generally it is required that the interactions within a community are dense while interactions between communities are sparse, but according to our new definition there is no interaction within a community since the population of EN species belonging to the same trophic level do not interact with each other. We then put forward a general multiobjective optimization model for discovering community structures from arbitrary multipartite ENs by introducing a competition mechanism among species from the viewpoint of graph theory. After a community structure is determined, we then adopt three typical perturbation strategies: (1) random order, (2) from the most important component to the least one, (3) the reverse way of (2), to evaluate the robustness of ENs to the species loss of community. Because in nature, species from different genera and families or even kingdoms may suffer from extinctions, in our study, different from most of the existing studies which only consider monotonous species (i.e., either plants or pollinators or predators) extinctions, communities at different trophic levels are given probabilities to go extinct. For an EN, we also investigate the relationships between the robustness of the network itself to species loss and that of its community composed network in which each node represents a community of the original network. Our experiments indicate that ecosystems are robust to random species loss of community but fragile to target ones, and that the robustness analysis of a large-scale ecosystem to species loss may be akin to that of its community composed network which is usually small in size.

## Results

### Community discovery analysis

**Figure 1:**
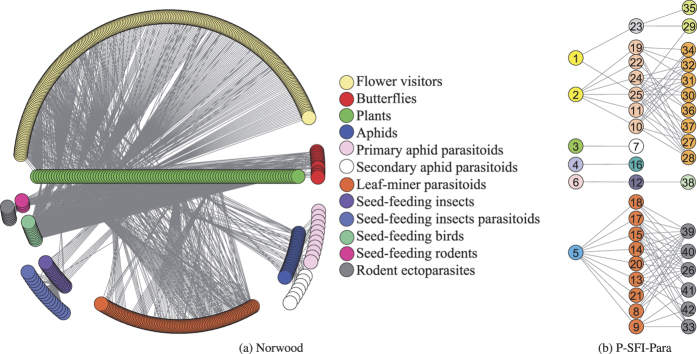
Community structures of the (**a**) Norwood network and (**b**) the P-SFI-Para network. Different colors denote different communities.

The Norwood Farm ecological network encompasses seven subnetworks. For the Norwood network, our model divides the whole network into 12 modules as shown in Fig. 1(a), which is exactly the same as that shown in ref. 16. As is annotated in the figure, each module denotes a certain kind of species.

For each subnetwork, our model divides it into smaller modules each of which only contains species from the same trophic level (See SI text). We believe that our discovered community structures are meaningful because a species module may, from the perspective of taxonomy, belong to the same genus, or family, or even order, and from the perspective of geography may belong to the same habitat. Here we take the P-SFI-Para network as an example to further elucidate this.

Figure 1(b) shows the obtained community structure of the P-SFI-Para network. The detailed information of the network is recorded in Table 1. From Fig. 1(b) and Table 1 we can see that, plants 1 and 2 belong to the same genus, while plants 3, 4, and 6 belong to different genera, and our model divides these three plant species as independent communities. Although plants 4 and 5 belong to the same genus, plant 5 is a generalist while plant 4 is a specialist. Consequently, to divide plants 4 and 5 into two communities is acceptable. Similar phenomena also happen to the seed-feeding insects and the seed-feeder parasitoids. For example, insects 23, 7, 16, and 12 are divided into independent communities. We may notice that insects 16 and 12 belong to the same family, however, insect 12 has parasitoid while insect 16 does not.

### Network robustness to community loss

**Figure 2:**
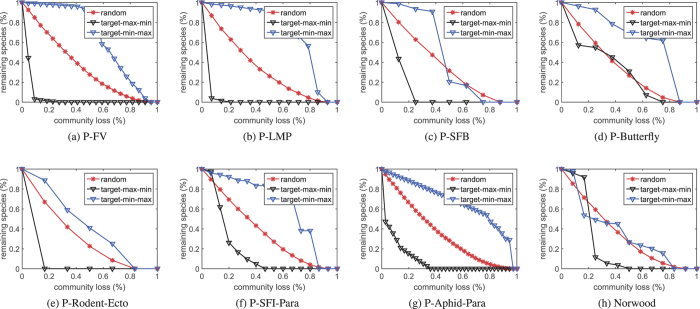
Proportion of the remaining species in the network when suffering from sequential species community loss. Each point is averaged over 10000 independent trials.

After the community structure is determined, we then test the network robustness to the species loss of community. The curves in Fig. 2 exhibit the proportions of the remaining species in the networks when suffering from sequential community loss with respect to three different extinction strategies.

In our experiments, a species is considered to be extinct when it has no interaction with other species. Table 2 records the *R*_*A*_ values of the eight networks. From Fig. 2 and Table 2, we can clearly see that the tested networks are robust to random community loss but fragile to target perturbation.

Although the conclusion we draw from the results in Fig. 2 and Table 2 is not surprising, we still would like to reclaim three important points of this work: (1) the newly defined community concept is meaningful and corresponds well to real situations; (2) the suggested multiobjective community detection model can be modified to fit with arbitrary networks; (3) multiple species (opposite to monotonous species like either plants or pollinators) should be taken into consideration when analyzing the robustness of ENs. With these in mind, we can better understand the dynamics of ENs.

### Robustness relationships between the original networks and the community composed networks

The robustness of an EN to the species loss of community is equivalent to the robustness of the weighted community composed network to species loss. Here, we both qualitatively and quantitatively investigate the relationships between the robustness of an EN and that of its unweighted community composed network both to species loss.

**Figure 3:**
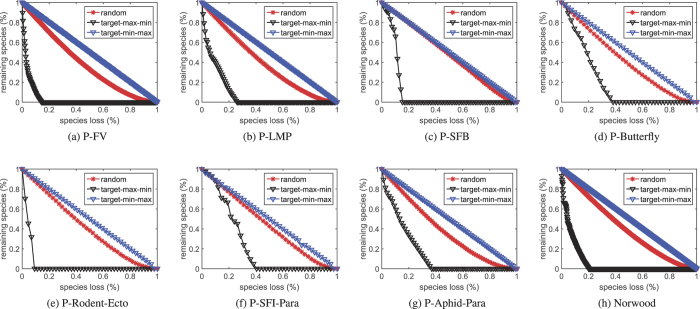
Proportion of the remaining species in the eight real-world networks when suffering from sequential species loss. Each point is averaged over 10000 independent trials.

**Figure 4:**
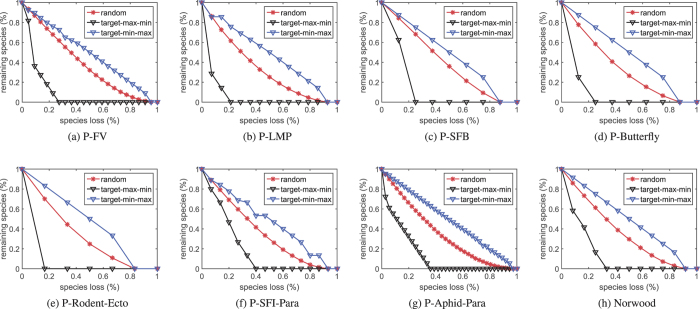
Proportion of the remaining species in the community composed networks of the eight real-world networks when suffering from sequential species loss. Each point is averaged over 10000 independent trials.

Figures 3 and 4 respectively exhibit the proportions of the remaining species in the original networks and the unweighted community composed networks when they are suffering from sequential species loss. It can be seen from the figures that for each network the curves in Fig. 3 and those in Fig. 4 show similar distributions.

We then use nonlinear fitting techniques to fit the curves in Fig. 4. After getting the corresponding coefficients, we use them to calculate the approximated curves (coefficients multiplied by the abscissae in Fig. 3). Finally, we calculate the errors between the approximated curves and those in Fig. 3. The sum of the squared errors (SSE) and the root mean square errors (RMSE) are recorded in Table 3.

It can be noticed from Table 3 that the errors are small except those marked in boldface. These results indicate that the robustness analysis of an EN (large in size) to species loss may be akin to that of its community composed network (small in size). As a matter of fact, for each network listed in Table 4, we have repeatedly tested the robustness of random-community (randomly generated communities) composed networks. However, we cannot obtain similar curves like those in Fig. 4.

## Discussion

This study is likely to offer a new perspective towards the understanding of the dynamics of ecosystems and thus helps to predict potential ecological crises and mitigate detrimental impacts on ecosystems and human beings.

In our study, for each EN we have decided to use the binary version of its adjacency matrix (the adjacency matrix of the converted network is weighted). The main reason is that the interaction strengths between species are mainly obtained by field observations and samplings which may not totally depict real situations. Indeed, we can test the weighted versions of the networks without making changes to the model and algorithm as used in the present experiments. However, the most important thing that we want to convey in this work is the new perspective towards the study of ecosystems’ dynamics, and by doing so, we would like to raise three fundamental questions which still need tremendous efforts to research.

- **Question 1** Is it possible to solve the species taxonomical problems from the perspective of complex network community detection?
- In our exp.eriments, our proposed model divides an EN into many communities and the division does make sense. However, not all the community structures always hold water as we do find a certain kind of community that contains species from different families or orders. For example, in Fig. 1(b), parasitoids 35 and 29, which come from different genera and families, have been classified into the same community, although this kind of classification is still explainable as we can notice from Table 1 that parasitoids 35 and 29 belong to the same order. The main reason is that only having the species interaction information would be insufficient. When modeling an EN, the interactions are mainly obtained by field observations and samplings. In real situations, species interactions may be time- and/or space-dependent. The work in ref. 32 indicated that time-spatial information of species may be a key component towards the understanding of species behavior. If we can incorporate more information to construct better network topology structures, or to perfect our proposed community detection model, or we even can use existing time-spatial community detection method like that in ref. 33, then we may solve the species taxonomical problems from the perspective of network science. Currently, however, our work seems to shed new light on this problem.
- **Question 2** How can we better define species competition?
- Competition is widespread not only in ecology but also in fields like social sciences and economics. In our study, we only consider competitions among species at the same trophic level. However, species from different trophic levels may also compete with each other so as to attain more niche resources. Besides, we define the species competition strengths based on the mutual neighborhoods. From the viewpoint of network science, species competition can be regarded as the similarity between two nodes. The authors in ref. 34 analyzed the stabilities of fifteen similarity measurements for bipartite recommendation networks. Each of those fifteen measurements can be a potential metric to quantify species competition. Consequently, whether our defined species competition mechanism really depicts true situations or not still needs to be further investigated from the perspective of complex ecosystems.
- **Question 3** Do there really exist certain relationships between the robustness of an EN and that of its community composed network?

In this study, from the experiments we empirically draw the conclusion that the robustness analysis of an EN which is usually large in size may be akin to that of its unweighted community composed network which is small in size. If the conclusion is true and can be proved theoretically, it would be of great importance to the understanding of the dynamics of complex systems.

## Materials and Methods

### Network data

Many researches on the robustness of ENs have been focused on bipartite networks like pollination networks and prey-predator food webs because these networks are well studied in ecology. As advocated in ref. 16, further attentions should be paid to analyzing large-scale ENs, e.g., a whole-farm scale EN.

In this study, we analyze the Norwood Farm ecological network which encompasses seven subnetworks. Network properties are given in Table 4. Detailed information can be found in refs 16 and 35.

### Multiobjective community discovery model for arbitrary unipartite graphs

Multiobjective community detection fulfils the definition of a network community, i.e., the similarities within a community are high while the similarities between different communities are low. Consequently, it is straightforward to establish the following multiobjective community detection model:


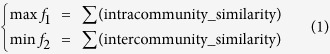


An efficient way to measure similarity is the interaction strength. Based on this, a general model for arbitrary unipartite graphs can be written as:


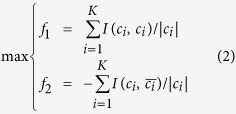


where *K* is the number of communities, *c*_*i*_ is the *i*-th community, and 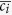 is the remaining part apart from *c*_*i*_. The symbol *I*(*a*, *b*) denotes the number of interactions between objects *a* and *b*, and |*c*_*i*_| is the size of *c*_*i*_. Equation (2) may have many variants such that it can fit for weighted, directed, or signed graphs.

Multiobjective community detection has two main advantages over other techniques. On one hand, it can overcome the resolution limit. On the other hand, it can facilitate multi-criteria intelligent decision making since each single run of a multiobjective community detection method will yield a set of solutions, each of which represents a community structure.

### Competition mechanism

**Figure 5:**
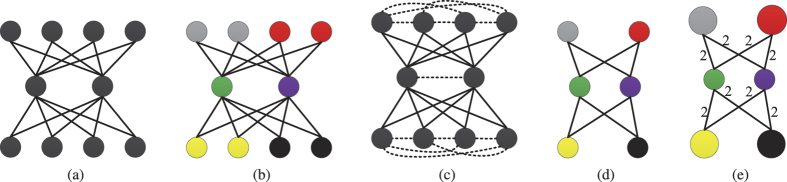
Network illustration of the introduced competition mechanisms. (**a**) a tripartite network. (**b**) a possible community structure in which different colors represent different communities. (**c**) the signed network after transformation. (**d**) the community composed network, i.e., each node represents a community in (**b**). (**e**) the weighted version of (**d**).

A graphical illustration of a *k*-partite EN when *k* = 3 is displayed in Fig. 5(a). According to our new definition of community, we attempt to obtain a community structure like the one shown in Fig. 5(b).

It can be noticed from Fig. 5(b) that within a community there is no interaction. Therefore, the model in equation (2) will not work (because 
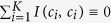
) unless there exist interactions within a community. In order to establish interactions between species within a community, we introduce the species competition mechanism.

Species competition is ubiquitous in ecology^36^,^37^. Many competition models like the famous Lotka-Volterra competition equations^38^ have been proposed. In this paper, we define the competition strength based on mutual neighborhood from the perspective of graph theory.

Given that *a*_*i*_ and *a*_*j*_ are the *i*-th and the *j*-th rows (species *i* and *j* come from the same trophic level) of the adjacency matrix *A* of an EN. Then the competition strength *Comp*(·) between species *i* and *j* is defined as *Comp*(*i*, *j*) = −1 · *a*_*i*_ · *a*_*j*_. Here we use −1 to distinguish competition interactions from real ones. With the introduced competition mechanism, the tripartite network shown in Fig. 5(a) is then converted into the signed network (Here we use dashed lines to denote competition interactions) as shown in Fig. 5(c), and the model in equation (2) will make sense. See SI text for details.

### Algorithm overview

By introducing the competition mechanism, a *k*-partite EN is converted into a unipartite signed EN. To discovery communities from the converted signed network, we modify equation (2) as follows:


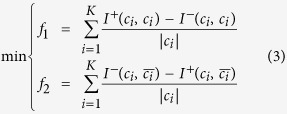


where the symbol *I*^+^(*a*, *b*) and *I*^−^(*a*, *b*), respectively, denotes the numbers of positive and negative interactions between communities *a* and *b*.

We utilize the multiobjective particle swarm optimization algorithm proposed in ref. 39 to optimize equation (3). We choose the Pareto solution that has the largest distance to the origin as the final community detection solution.

When testing the robustness of ENs to community loss, the importance of a community *c*_*i*_ is defined as 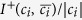. In the literature, many metrics have been proposed to measure the robustness of networks to perturbations, such as those listed in ref. 40, the R50 index^41^, the area-based index *R*_*A*_^16^, and the node robustness index^12^. In our experiments, the *R*_*A*_ metric is adopted because it is well suited. See SI text for details.

## Additional Information

**How to cite this article**: Cai, Q. and Liu, J. The robustness of ecosystems to the species loss of community. *Sci. Rep.* **6**, 35904; doi: 10.1038/srep35904 (2016).

**Publisher’s note:** Springer Nature remains neutral with regard to jurisdictional claims in published maps and institutional affiliations.

## Supplementary Material

Supplementary Information

## Figures and Tables

**Table 1:** Detailed information for the P-SFI-Para network.

Node ID	Species	Taxonomic Name	Genus	Family	Order
1, 2	plant	Cirsium arvense (vulgare)	Cirsium	Asteraceae	Asterales
3	plant	Crataegus monogyna	Crataegus	Rosaceae	Rosales
4	plant	Trifolium dubium	Trifolium	Fabaceae	Fabales
6	plant	Vicia sativa	Vicia	Fabaceae	Fabales
5	plant	Trifolium pratense/repens	Trifolium	Fabaceae	Fabales
23	SFI	Terellia ruficauda	Terellia	Tephritidae	Diptera
7	SFI	Blastodacna hellerella	Blastodacna	Elachistidae	Lepidoptera
12	SFI	Oxystoma pomonae	Oxystoma	Apionidae	Coleoptera
13-18	SFI	—	Protapion	Apionidae	Coleoptera
35-38	Para	—	Pteromalus	Pteromalidae	Hymenoptera
29	Para	Bracon praecox	Bracon	Braconidae	Hymenoptera
31, 32	Para	Eurytoma sp A (B)	Eurytoma	Eurytomidae	Hymenoptera
41, 42	Para	—	Trichomalus	Pteromalidae	Hymenoptera
40	Para	Triaspis floricola	Triaspis	Braconidae	Hymenoptera

**Table 2:** Robustness of the eight networks to community loss.

(*R*_*A*_ index)	random	target-max-min	target-min-max
P-FV	0.3576 ± 0.1129	0.0462 ± 0	0.6602 ± 0.0005
P-LMP	0.3419 ± 0.1642	0.0395 ± 0	0.7215 ± 0.0015
P-SFB	0.3711 ± 0.1617	0.1170 ± 0	0.4631 ± 0
P-Butterfly	0.3425 ± 0.1589	0.3065 ± 0	0.6413 ± 0.0015
P-Rodent-Ecto	0.3177 ± 0.1380	0.0833 ± 0	0.4394 ± 0.0189
P-SFI-Para	0.3625 ± 0.0933	0.1778 ± 0	0.6524 ± 0
P-aphid-Para	0.3503 ± 0.0961	0.0830 ± 0.0007	0.6702 ± 0.0105
Norwood	0.3429 ± 0.1499	0.2155 ± 0	0.3565 ± 0.0019

The *R*_*A*_ values are averaged over 10000 independent trials.

**Table 3:** Nonlinear fitting for the curves in Fig. 3.

(*R*_*A*_ index)	random		target-max-min		target-min-max	
P-FV	SSE	0.0269	SSE	**4.9541**	SSE	0.0318
	RMSE	0.0097	RMSE	0.1309	RMSE	0.0105
P-LMP	SSE	0.1572	SSE	0.3892	SSE	0.1319
	RMSE	0.0345	RMSE	0.0543	RMSE	0.0316
P-SFB	SSE	0.3352	SSE	0.5454	SSE	0.0234
	RMSE	0.0651	RMSE	0.0831	RMSE	0.0172
P-Butterfly	SSE	0.2994	SSE	**0.7346**	SSE	0.0418
	RMSE	0.0834	RMSE	0.1370	RMSE	0.0312
P-Rodent-Ecto	SSE	0.3977	SSE	0.1489	SSE	0.2061
	RMSE	0.0940	RMSE	0.0575	RMSE	0.0677
P-SFI-Para	SSE	0.2494	SSE	0.2541	SSE	0.0210
	RMSE	0.0762	RMSE	0.0769	RMSE	0.0221
P-aphid-Para	SSE	0.1218	SSE	0.1287	SSE	0.0026
	RMSE	0.0398	RMSE	0.0409	RMSE	0.0058
Norwood	SSE	**1.3633**	SSE	**1.1438**	SSE	0.0995
	RMSE	0.0493	RMSE	**0.3225**	RMSE	0.0133

**Table 4:** Parameters of the eight tested ecological networks.

Network	#Node	#Edge	type	*k*
P-FV	47–241	501	Mutualistic	2
P-LMP	35–96	219	Trophic	2
P-SFB	66–12	434	Trophic	2
P-Butterfly	26–16	76	Mutualistic	2
P-Rodent-Ecto	32-4-8	96	Trophic-Parasitic	3
P-SFI-Para	6-19-17	91	Trophic	3
P-aphid-Para	30-28-18	84	Trophic	3
Norwood	560	1501	Mixed	>3

P–plants, FV–flower visitors, LMP–leaf- miner parasitoids, SFB–seed-feeding birds, ecto–rodent ectoparasites, SFI–seed-feeding insect, para–parasitoids.
