# Supplementary material for: The robustness of ecosystems to the species loss of community
Source: Sci Rep. 2016 Oct 27;6:35904. doi: 10.1038/srep35904 (PMC5082364; doi:10.1038/srep35904)
Supplement: Supplementary Information [file srep35904-s1.pdf]

The robustness of ecosystems to the species loss of community

Supporting Information

Qing Cai and Jiming Liu

Department of Computer Science, Hong Kong Baptist University, Kowloon Tong KLN, Hong Kong

SI Text

Studies on the Underlying Mechanisms That Affect the Stabilities of Ecological Networks

Because structures always affect functions, consequently, it is of great value to analyze the structural properties of ecological networks (ENs) [1]. A long-standing quest of ecology is about the stabilities of ENs. The first challenging question that needs to be answered is what factors affect the stabilities of ENs. A problem that besets ecologists for nearly a century is about the complexity and stability of ENs. The influential work of Robert May in Ref. [2] unequivocally pointed out that complexity destabilizes a system. May's theory has been a theoretical dogma for over two decades until McCann et al. [3] found evidence that complexity promotes stability [4]. However, the debate between complexity and stability is still continuing [5, 6, 7, 8]. No matter whether complexity begets stability or not, it is widely accepted that real-world ENs are nonrandom and have certain structure patterns to increase their persistence [9, 10]; for example, the work in Ref. [11] discovered that intervality and broad degree distributions contribute to the stability of food webs, and food webs' functional group diversity increases with modularity [12].

One well studied topological characteristic of complex networks is the degree distribution, e.g., the scale-free (SF) [13] and small-world (SW) [14] properties. Ecologists found that some ENs display SF and/or SW behaviors [15], which suggests that ENs are robust to random species loss but fragile to target attacks [16]. However, most ENs do not display SF and/or SW behaviors [17]. Another well studied characteristic of ENs is the link distribution which is commonly described in terms of "connectance" [18], "nestedness" [19] and "compartmentalization" [20] or "modularity" [21]. The research in Ref. [22] found that connectance has a positive effect on the robustness of food webs. The findings in Refs. [23] and [24] respectively showed that mutualistic networks tend to be nested whereas trophic networks to be modular. However, it has been proved that nestedness may be a ghost in ENs [25]. A very recent work in Ref. [26] further pointed out that whether a network is significantly modular or nested depends on the null model one used. It should be noted that both nestedness and modularity are architectural patterns to increase the persistence of species. It has been discovered that for different types of ENs, the structural patterns that affect the network robustness are different. For trophic networks, the patterns exhibit week connectance and modularity [27]. On the contrary, for mutualistic networks the patterns are of the types of high connectance and nestedness [1] or the abundance of the rarest species [28]. It it however interesting to note that there is no significant correlation between nestedness and modularity [29, 30].

Algorithm Implementation

For a given ecological network G, in order to investigate its robustness to the species loss of community, we first have to develop a method to divide the network into modules. Due to the special nature of network G (k-partite) and the novel notion of species community as we have defined, we introduced the species competition mechanism whereby network G is converted into a signed network SG. Afterward, we put forward a multiobjective optimization model to detect community structures from SG. The optimization algorithm we used in this paper is the MODPSO algorithm proposed in our previous work. We

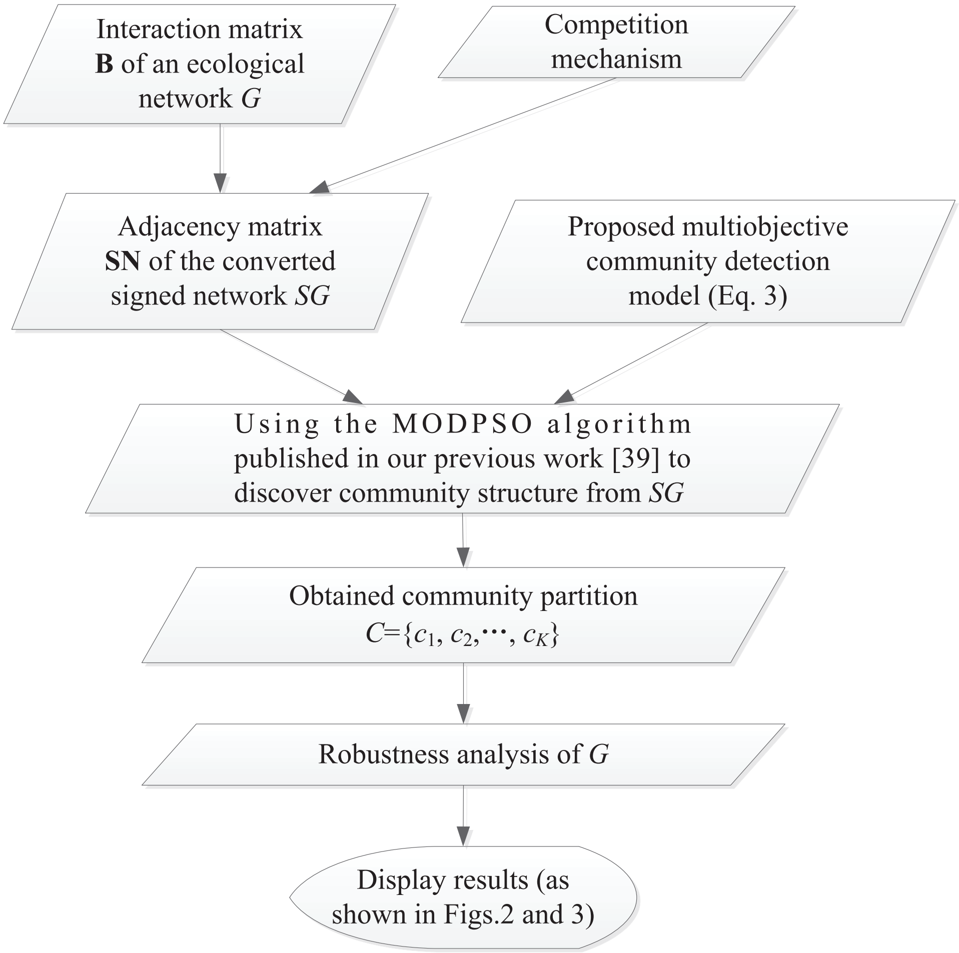

Figure 1: A flowchart of the whole process of the work.

use the detected community structure of *SG* as the network partition of *G*. Then the next step is to analyze the robustness of *G*. The flowchart of how our work is carried out is presented in Fig. 1. In the following, we will elucidate in detail the key components of how our work is carried out.

Illustration to Competition Calculation

Species may compete with each other in order to obtain more niche resources. However, species competition is not reflected in a k-partite ecological network. The main purpose of our work is to investigate the robustness of ecological networks to the species loss of community. For an ecological network, we define a community as a population of species that belong to the same trophic level, i.e., a set of vertices that come from the same partite set. The requirement for a community is that the similarities within a community are high while between different communities the similarities are low. For a k-partite ecological network, an edge only exists between two vertices that come from different partite sets. In our work we introduced the species competition strategy. A graphical illustration on calculating the species competition is given in Fig. 2.

In Fig. 2, **B** is the binary interaction or contact matrix of the original bipartite network *G*, **A** and **SN** are respectively the adjacency matrices of network *G* and the converted signed network *SG*, and the vector $\mathbf{a}_i$ is the i-th row of **A**. The competition strength $Comp(i, j)$ between species i and j is defined as the negative value of the dot product of $\mathbf{a}_i$ and $\mathbf{a}_j$.

By introducing the species competition strategy we then build the relationships between vertices within the same partite set, and as a consequence the original network *G* is then converted into a signed network *SG* which can be divided into

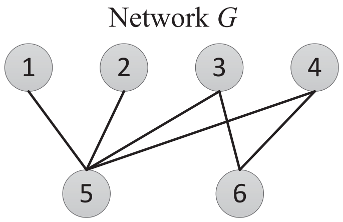

$$\mathbf{B} = \begin{bmatrix} 1 & 0 \\ 1 & 0 \\ 1 & 1 \\ 1 & 1 \end{bmatrix} \Rightarrow \mathbf{A} = \begin{bmatrix} \mathbf{0} & \mathbf{B} \\ \mathbf{B}^{\mathrm{T}} & \mathbf{0} \end{bmatrix} = \begin{bmatrix} 0 & 0 & 0 & 0 & 1 & 0 \\ 0 & 0 & 0 & 0 & 1 & 0 \\ 0 & 0 & 0 & 0 & 1 & 1 \\ 0 & 0 & 0 & 0 & 1 & 1 \\ 1 & 1 & 1 & 1 & 0 & 0 \\ 0 & 0 & 1 & 1 & 0 & 0 \end{bmatrix}$$

$$Comp(i, j) = -1 \cdot \mathbf{a}_i \cdot \mathbf{a}_j$$

$$Comp(1,2) = -1 \cdot \mathbf{a}_1 \cdot \mathbf{a}_2 = \text{-}1 \cdot [0\,0\,0\,0\,1\,0] \cdot [0\,0\,0\,0\,1\,0]^{\mathrm{T}} = -1$$
$$Comp(1,3) = -1 \cdot \mathbf{a}_1 \cdot \mathbf{a}_3 = -1$$
$$Comp(1,4) = -1 \cdot \mathbf{a}_1 \cdot \mathbf{a}_4 = -1$$
$$Comp(5,6) = -1 \cdot \mathbf{a}_5 \cdot \mathbf{a}_6 = \text{-}1 \cdot [1\,1\,1\,1\,0\,0] \cdot [0\,0\,1\,1\,0\,0]^{\mathrm{T}} = -2$$

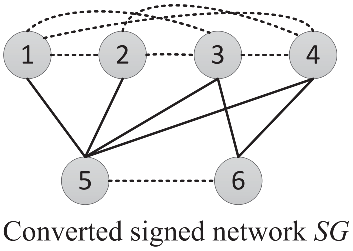

$$\mathbf{SN} = \begin{bmatrix} Comp_{4\times 4} & \mathbf{B} \\ \mathbf{B}^{\mathrm{T}} & Comp_{2\times 2} \end{bmatrix} = \begin{bmatrix} 0 & -1 & -1 & -1 & 1 & 0 \\ -1 & 0 & -1 & -1 & 1 & 0 \\ -1 & -1 & 0 & -2 & 1 & 1 \\ -1 & -1 & -2 & 0 & 1 & 1 \\ 1 & 1 & 1 & 1 & 0 & -2 \\ 0 & 0 & 1 & 1 & -2 & 0 \end{bmatrix}$$

Figure 2: Illustration to calculate the species competition.

communities as we defined. It should be noted that the interaction matrix **B** in Fig. 2 may have another form (its transpose form), but it does not matter.

Discovered Community Structures

The discovered community structures of the seven subnetworks of the Norwood Farm ecological network are presented in Figs. 3-9.

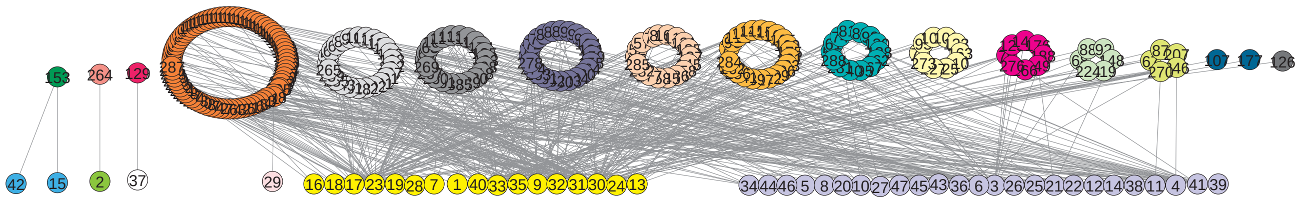

Figure 3: Community structure of the P-FV network

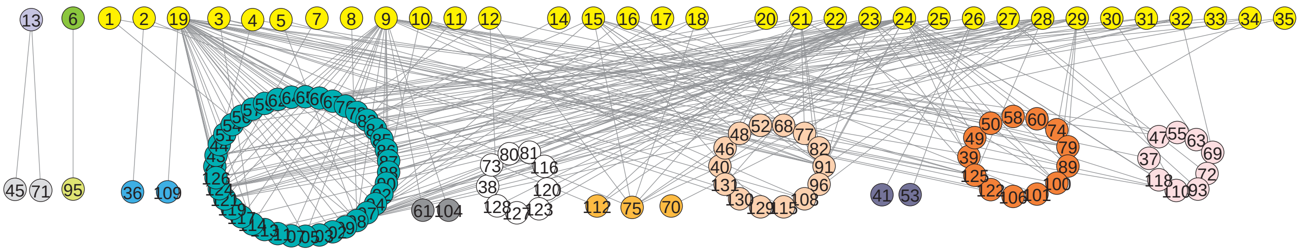

Figure 4: Community structure of the P-LMP network

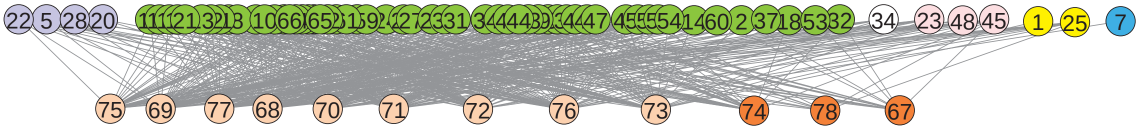

Figure 5: Community structure of the P-SFB network

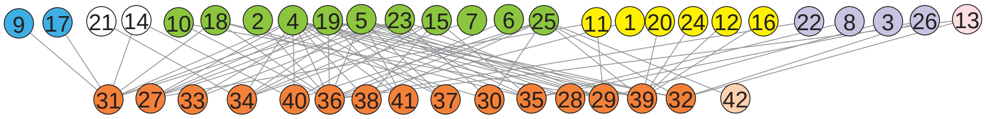

Figure 6: Community structure of the P-Butterfly network

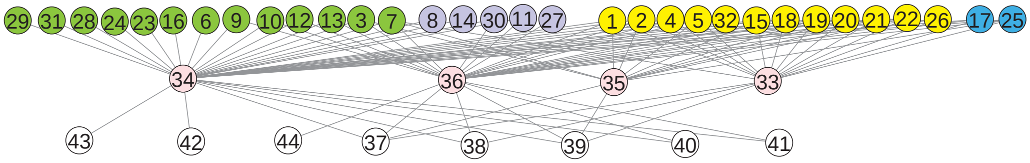

Figure 7: Community structure of the P-Rodent-Ecto network

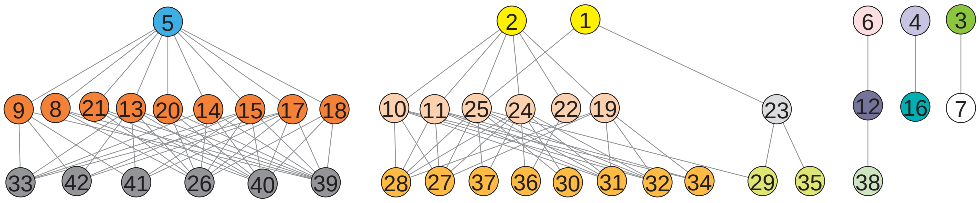

Figure 8: Community structure of the P-SFI-Para network

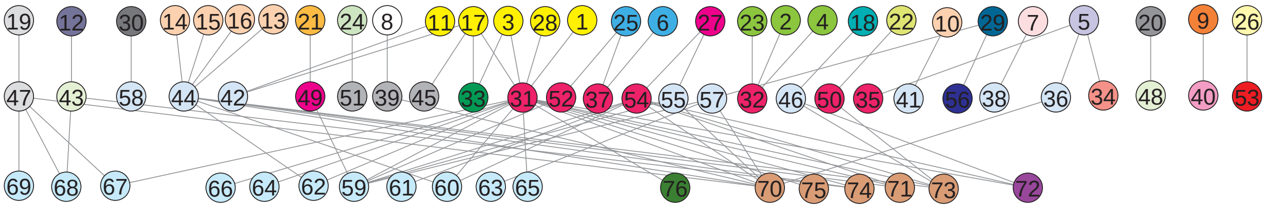

Figure 9: Community structure of the P-aphid-Para network

In Figs. 3-9 (drawn using the Pajek software), different colors represent different species communities. In each of the figures, all of the vertices on the upper (middle or bottom) side denote a certain kind of species; for example, in Fig. 3, all of the vertices on the upper side denote the flower visitors of plants. Because each of the P-FV and the P-LMP networks has over a hundred vertices, we therefore used circles in Figs. 3 and 4 to better display the network structures.

In the main paper, we analyzed the community structure of the P-SFI-Para network in detail. We showed that the detected community structure is meaningful from the perspective of species taxonomy. However, one thing should be kept in mind that there is no direct correlation between the detected network community structure and species taxonomy. The detected structure may shed light on species taxonomy, but it does not solve the taxonomical problem. On one hand, if we want to solve the taxonomical problem from the viewpoint of network clustering, then we may need hierarchical clustering technique; on the other hand, the purpose of this work is not dedicated to species taxonomy.

Illustration to Importance Calculation

In the experiments we adopted three perturbation strategies (random, target-max-min, and target-min-max) to investigate the robustness of ecological networks to perturbations. When testing the robustness of ecological networks to the species loss of community, we have to rank the communities according to their importances. Fig. 10 presents a graphical illustration to calculate the community importance.

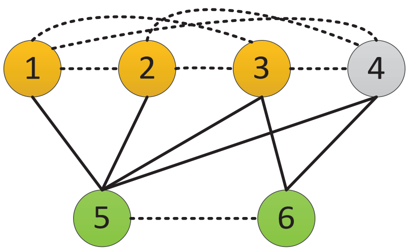

$$\text{community } C = \{c_1, c_2, c_3\}$$
$$c_1 = \{1, 2, 3\},\ c_2 = \{5, 6\},\ c_3 = \{4\}$$
$$|c_1| = 3,\ |c_2| = 2,\ |c_3| = 1$$
$$\text{importance}(c_i) = I^+(c_i, \bar{c}_i) / |c_i|$$
$$\text{importance}(c_1) = I^+(c_1, \bar{c}_1) / |c_1| = 4/3 = 1.333$$
$$\text{importance}(c_2) = I^+(c_2, \bar{c}_2) / |c_2| = 6/2 = 3$$
$$\text{importance}(c_3) = I^+(c_3, \bar{c}_3) / |c_3| = 2/1 = 2$$

Figure 10: An illustration on calculating the community importance.

In Fig. 10, C is a possible community partition with three communities, i.e., c_1, c_2 and c_3. Community c_1 contains nodes 1, 2 and 3, c_2 contains nodes 5 and 6, c_3 contains node 4.

When testing the robustness of ecological networks to the sequential species loss, we utilize the degree centrality metric to measure the importances of species. In the case of two or more communities (species) having the same importance, in the experiments we randomly rank them.

Network Robustness Analysis

In Fig. 1, the robustness analysis of G mainly contains two parts, i.e., the robustness of G to sequential species community loss (as shown in Fig. 2 of main paper) and the robustness of G to sequential species loss (as shown in Fig. 3 of main paper). In Fig. 11 we give a graphical illustration of the former one.

In Fig. 11, we suppose that the community partition is $C = \{\ \{1,2,3\}, \{4\}, \{5,6\}\ \}$ and the sequential community loss order is $\{1,2,3\} \rightarrow \{4\} \rightarrow \{5,6\}$. After removing community $\{1,2,3\}$ we have three species left. When we continue to remove community $\{4\}$, no species is left (species 5 and 6 are regarded to go extinct because they have no suppliers). Because the order of community loss depends on the perturbation strategies, and for a certain strategy the order also may be different, consequently we carry out the robustness analysis for 10000 independent times and average the results.

References

[1] R. P. Rohr, S. Saavedra, J. Bascompte, On the structural stability of mutualistic systems, Science 345 (2014) 1253497.

[2] R. M. May, Will a large complex system be stable?, Nature 238 (1972) 413–414.

[3] K. McCann, A. Hastings, G. R. Huxel, Weak trophic interactions and the balance of nature, Nature 395 (1998) 794–798.

[4] G. A. Polis, Ecology: Stability is woven by complex webs, Nature 395 (1998) 744–745.

[5] K. S. McCann, The diversity–stability debate, Nature 405 (2000) 228–233.

[6] A. R. Ives, S. R. Carpenter, Stability and diversity of ecosystems, Science 317 (2007) 58–62.

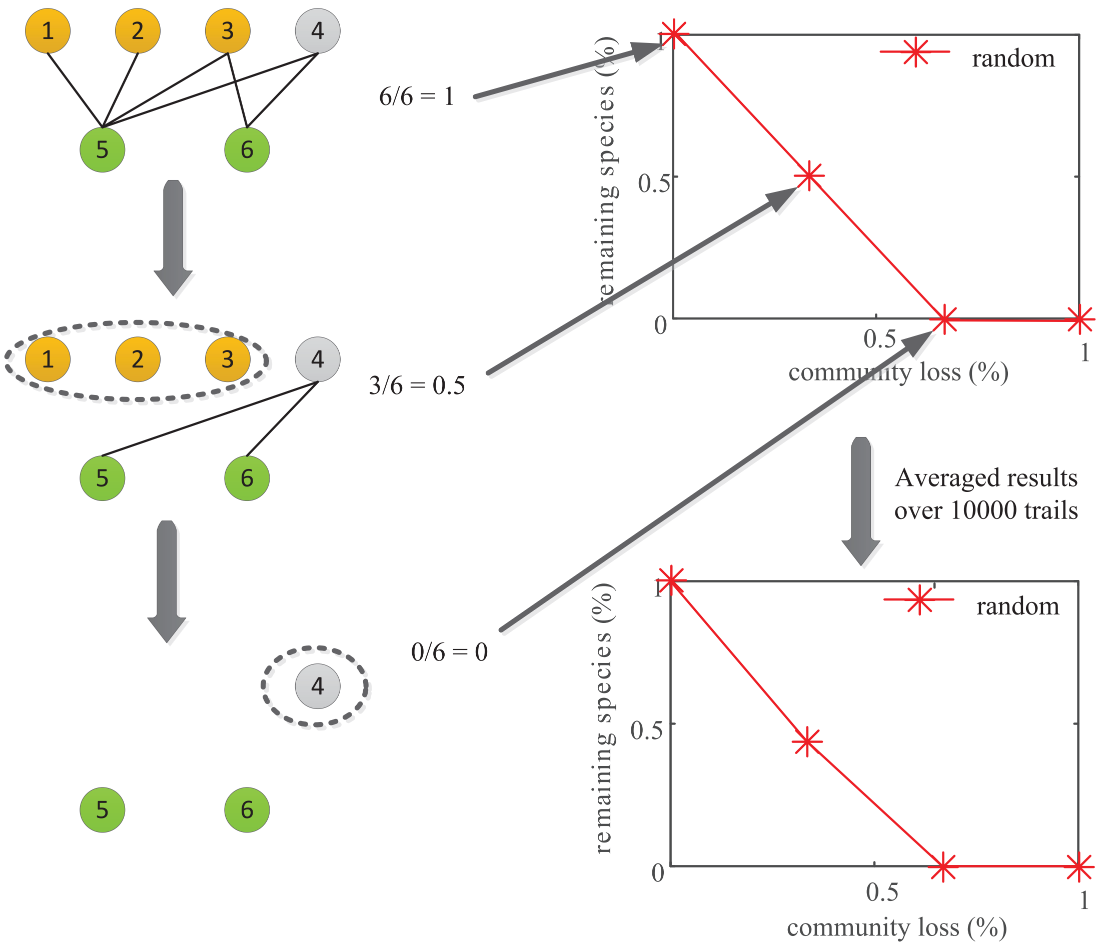

Figure 11: A graphical illustration of the analysis of network robustness to random community loss.

[7] I. L. Boyd, The art of ecological modeling, Science 337 (2012) 306–307.

[8] S. Allesina, S. Tang, Stability criteria for complex ecosystems, Nature 483 (2012) 205–208.

[9] J. M. Montoya, S. L. Pimm, R. V. Solé, Ecological networks and their fragility, Nature 442 (2006) 259–264.

[10] J. Bascompte, Structure and dynamics of ecological networks, Science 329 (2010) 765–766.

[11] S. Allesina, J. Grilli, G. Barabás, S. Tang, J. Aljadeff, A. Maritan, Predicting the stability of large structured food webs, Nat. Commun. 6 (2015) 1–6.

[12] D. Montoya, M. Yallop, J. Memmott, Functional group diversity increases with modularity in complex food webs, Nat. Commun. 6 (2015) 1–9.

[13] A.-L. Barabási, R. Albert, Emergence of scaling in random networks, Science 286 (1999) 509–512.

[14] D. J. Watts, S. H. Strogatz, Collective dynamics of ‘small-world’ networks, Nature 393 (1998) 440–442.

[15] J. M. Montoya, R. V. Solé, Small world patterns in food webs, J. Theor. Biol. 214 (2002) 405–412.

[16] R. V. Sole, M. Montoya, Complexity and fragility in ecological networks, Proc. Roy. Soc. Lond. B. Biol. Sci. 268 (2001) 2039–2045.

[17] R. J. Williams, E. L. Berlow, J. A. Dunne, A.-L. Barabási, N. D. Martinez, Two degrees of separation in complex food webs, Proc. Natl. Acad. Sci. USA 99 (2002) 12913–12916.

[18] J. Cohen, F. Briand, C. Newman, Community food webs: data and theory, Vol. 20, Springer Science & Business Media, 2012.

[19] M. Almeida-Neto, P. Guimaraes, P. R. Guimarães, R. D. Loyola, W. Ulrich, A consistent metric for nestedness analysis in ecological systems: reconciling concept and measurement, Oikos 117 (2008) 1227–1239.

[20] R. Guimera, D. Stouffer, M. Sales-Pardo, E. Leicht, M. Newman, L. Amaral, Origin of compartmentalization in food webs, Ecology 91 (2010) 2941–2951.

[21] M. Girvan, M. E. J. Newman, Community structure in social and biological networks, Proc. Natl. Acad. Sci. USA 99 (2002) 7821–7826.

[22] J. A. Dunne, R. J. Williams, N. D. Martinez, Network structure and biodiversity loss in food webs: robustness increases with connectance, Ecol. Lett. 5 (2002) 558–567.

[23] J. Bascompte, P. Jordano, C. J. Melián, J. M. Olesen, The nested assembly of plant-animal mutualistic networks, Proc. Natl. Acad. Sci. USA 100 (2003) 9383–9387.

[24] E. Thébault, C. Fontaine, Stability of ecological communities and the architecture of mutualistic and trophic networks, Science 329 (2010) 853–856.

[25] P. P. Staniczenko, J. C. Kopp, S. Allesina, The ghost of nestedness in ecological networks, Nat. Commun. 4 (2013) 273–275.

[26] S. Nuwagaba, C. Hui, The architecture of antagonistic networks: Node degree distribution, compartmentalization and nestedness, Comput. Ecol. Softw. 5 (2015) 317–327.

[27] D. B. Stouffer, J. Bascompte, Compartmentalization increases food-web persistence, Proc. Natl. Acad. Sci. USA 108 (2011) 3648–3652.

[28] S. Suweis, F. Simini, J. R. Banavar, A. Maritan, Emergence of structural and dynamical properties of ecological mutualistic networks, Nature 500 (2013) 449–452.

[29] J. M. Olesen, J. Bascompte, Y. L. Dupont, P. Jordano, The modularity of pollination networks, Proc. Natl. Acad. Sci. USA 104 (2007) 19891–19896.

[30] M. A. Fortuna, D. B. Stouffer, J. M. Olesen, P. Jordano, D. Mouillot, B. R. Krasnov, R. Poulin, J. Bascompte, Nestedness versus modularity in ecological networks: two sides of the same coin?, J. Anim. Ecol. 79 (2010) 811–817.
